# First case report of *Cryptococcus laurentii* knee infection in a previously healthy patient

**DOI:** 10.1186/s12879-020-05401-z

**Published:** 2020-09-17

**Authors:** Hetao Huang, Jianke Pan, Weiyi Yang, Jiongtong Lin, Yanhong Han, Kai Lan, Lingfeng Zeng, Guihong Liang, Jun Liu

**Affiliations:** 1 Second School of Clinical Medicine, Guangzhou University of Chinese Medicine, Guangzhou, China; 2 Department of Sports Medicine, Second Affiliated Hospital of Guangzhou University of Chinese Medicine, Guangdong Provincial Hospital of Chinese Medicine, Guangzhou, China; 3 Department of Clinical Laboratory, Second Affiliated Hospital of Guangzhou University of Chinese Medicine, Guangdong Provincial Hospital of Chinese Medicine, Guangzhou, China; 4 Bone and Joint Research Team of Degeneration and Injury, Guangdong Provincial Academy of Chinese Medical Sciences, Guangzhou, China

**Keywords:** Fungal infection, *Cryptococcus laurentii*, Case report

## Abstract

**Background:**

The purpose of this case report was to report a case of *Cryptococcus laurentii* infection in the left knee of a previously healthy 29 year old male patient.

**Case presentation:**

After an initial misdiagnosis and 7 months of failed treatment, the patient received nearly a month of treatment with voriconazole (200 mg IV q12 h) and knee irrigation with amphotericin B until the infection was controlled. The treatment continued with fluconazole for nearly 7 months and approximately 5 weeks of antibiotic treatment for a skin bacterial coinfection. In the end, the patient’s symptoms disappeared completely, the left knee recovered well, and there was no recurrence of infection.

**Conclusion:**

The key points of successful treatment in this case were the thorough debridement, the adequate course of knee irrigation with antifungal drugs and more than 6 months of oral antifungal drugs that were able to eradicate the infection.

## Background

Compared with those in high-income countries, musculoskeletal infections including acute osteoarticular infections in children, osteomyelitis, septic arthritis and osteomyelitis with adjacent septic arthritis in developing countries are more common [1]. The *Staphylococcus* genus remains the most frequent cause of musculoskeletal infections and is responsible for more than 58% of the osteoarticular infections. Coagulase-negative staphylococci were the most frequently isolated bacteria [2]. In recent years, more and more studies have shown that fungal musculoskeletal infections occur in immunocompromised and previously healthy patients. Fungal bone and joint infection is an uncommon but devastating infection, which requires surgery and long-term antifungal therapy. The mostly commonly reported genera are *Candida* and *Aspergillus, *[3] but a wide array of other opportunistic fungi, including *Fusarium* spp., *Scedosporium* spp., Mucorales, and dematiaceous molds, have been reported [4]. Among the fungal infections, cryptococcal infection is more serious, which should be paid attention to. There are two main types of *cryptococcosis*, namely *Cryptococcus neoformans* and *Cryptococcus neoformans*. The incidence of infection due to these organisms has increased during the past 40 years, with *Cryptococcus laurentii* and *Cryptococcus albidus* being responsible for 80% of all cases of *Cryptococcus *[5]. *Cryptococcus laurentii* infections have been reported as central nervous system infections, bloodstream infections, pulmonary infections and infections in other body sites [6–11], but a patient with osteoarthritis and a *Cryptococcus laurentii* infection has not been reported so far. We report a case of fungal arthritis due to *Cryptococcus laurentii* in an previously healthy patient at our institution.

## Case Presentation

A 29-year-old male was admitted on November 4th, 2016, due to recurring left knee pain, swelling and limited activity over 7 months. The patient had a history of being scratched by plants in March 2016 and received a penetrating wound from a thorn on the posterolateral side of his left knee and experienced symptoms of pain, warmth and swelling in the left knee joint. Treatments including antibiotics (cefazolin sodium and levofloxacin), intra-articular puncture with knee fluid aspiration and steroidal injection were performed repeatedly at a local hospital, and the symptoms were recurrent. The patient denied any prior physical diseases or infectious diseases including tuberculosis.

**Fig. 1:**
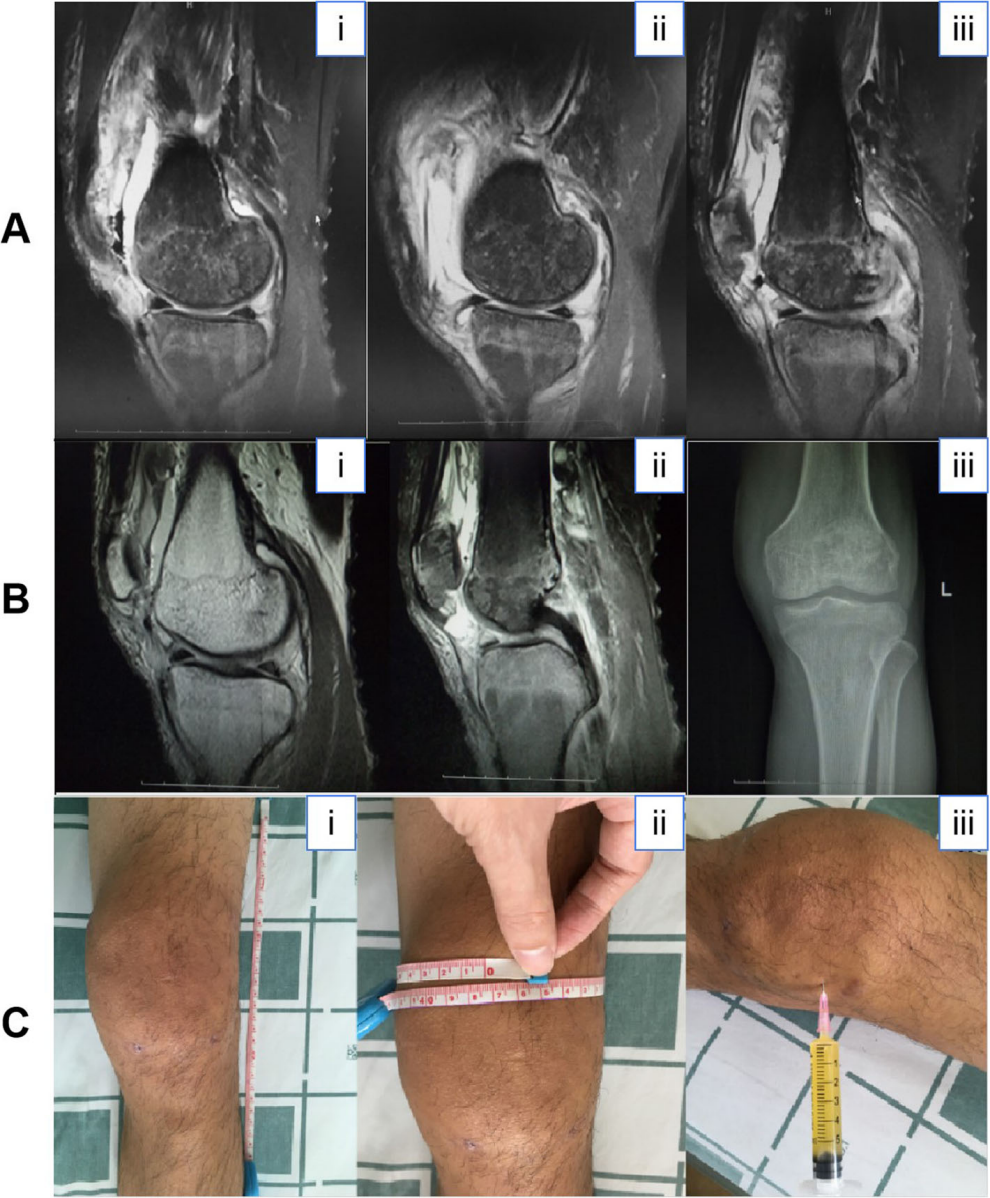
**a**. MR imaging indicated bone marrow edema of the proximal tibia, distal femur and patella, posterior horn tears of the medial and lateral meniscus (III°), and joint effusion, all of which confirmed the clinical diagnosis of purulent arthritis. **b**(i,ii). MR imaging after knee arthroscopic surgery revealed that the joint swelling was reduced, the proximal tibia edema was improved, and the left lateral meniscus was repaired, while the other conditions were similar to those observed in the first MR images. **b**(iii). Left knee DR revealed swelling of the soft tissue of the left knee joint, and the bone was loose, indicative of osteoporosis. **c**. The left knee was swollen; the longitudinal range of swelling was 29 cm, and the knee circumference at the most swollen part was 47.4 cm. Purulent fluid was withdrawn from the left knee

The physical examination showed the left knee was moderately swollen without effusion and the local skin temperature was slightly increase. The range of motion was − 3° to 90°. The floating patella test was positive while the drawer test and patella grinding test were negative. And these tests indicate presence of a knee joint effusion in the absence of meniscal tear. The MR imaging indicated bone marrow edema of the proximal tibia, distal femur and patella, posterior horn tears of the medial and lateral meniscus (III°), and joint effusion that confirmed the clinical diagnosis of purulent arthritis (Fig. 1.a). The laboratory examination results showed that inflammatory markers were elevated: the erythrocyte sedimentation rate was 77 mm/h and the concentration of high-sensitivity C-reactive protein was 61 mg/dL. There is no obvious abnormality in the patient’s immune test results, such asabsolute lymphocyte counts, HIV status and hepatitis serologies.

Surgical of the left knee with a synovectomy and meniscus repair under arthroscopy was performed on November 7th, 2016. Intraoperatively, a large amount of white pus was observed in the knee cavity, the synovium was massively hypertrophic and hyperemic, and the white area of the posterior horn of the lateral meniscus exhibited longitudinal tearing and was unstable. The medial meniscus, the crucial ligaments and the articular surfaces appeared normal. The pus and synovium samples were sent to be cultured for bacteria, fungus and tuberculosis. During the operation, the hyperplastic synovium was resected, a meniscus plasty was performed, the knee cavity was irrigated, and a drainage tube was placed.

Postoperatively, the left knee was irrigated with Gentamicin sulfate and other routine treatments were carried out for 5 days. After arthroscopic surgery, repeat MR imaging revealed joint swelling was reduced, the proximal tibia edema was improved, and the left lateral meniscus was repaired, while other conditions were similar to those in the first MR images (Fig. 1.b.i and ii). The culture results revealed an elevated nucleated cell count (4800 × 10^6^/L) in the synovial fluid, and the results of bacteria and tuberculosis cultures were negative. The patient recovered routinely and was discharged on November 15th, 2016.

At the same time, the laboratory was still in the cultivation and identification of fungi.The process of fungal culture and identification is as follows. Two sabourand’s medium without antibiotics were used for fungal culture at 37 °C and 25 °C. The Matrix-Assisted Laser Desorption/Ionization Time of Flight Mass Spectrometry (MALDI-TOF MS) technology provided by VITEK® ms of biomerie, France, was used for strain identification. Nearly fifteen days later, the clinical laboratory reported that the culture result of the patient’s operative sample revealed a *Cryptococcus laurentii* infection. The patient was immediately contacted and admitted again on December 12th, 2016, to treat the left knee fungal infection.

A physical examination showed the patient’s left knee was moderately swollen and warm, and more than 5 ml of yellow purulent fluid was withdrawn (Fig. 1.c). The laboratory examination results showed that the erythrocyte sedimentation rate was 24 mm/h, and level of high-sensitivity C-reactive protein was normal. Left knee digital radiography (DR) revealed obvious swelling of the soft tissue, and the bone was loose, indicating osteoporosis (Fig. 1.b.iii), bearing in mind that osteoporosis is associated with a deficiency in bearing the heavy load of the lower limbs.

According to the clear diagnosis of fungal infection and drug sensitivity, we held a consultation with specialists from the pharmacy department and made an initial treatment plan including 200 mg of intravenous (IV) voriconazole every 12 h (q12 h; 400 mg q12 h on the first day), debridement and irrigation of the left knee with amphotericin B until the infection was controlled and 400 mg of fluconazole per os (PO) q24 h maintained for 6 months to eradicate the infection.

**Fig. 2:**
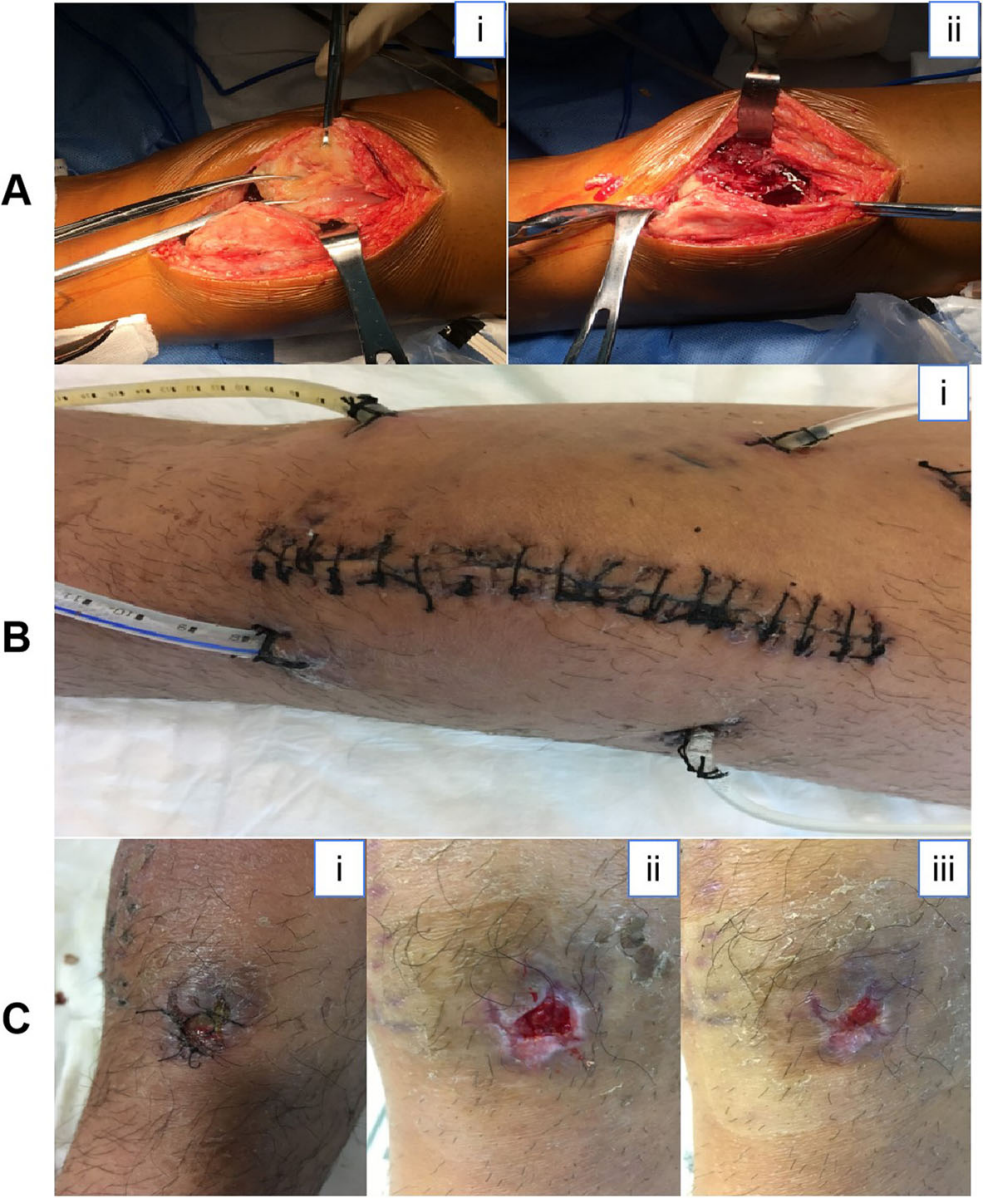
**a**. Intraoperative manifestations of knee joint. **b**. Yellow, purulent liquid was drained from the medial drainage tube, and it became obstructed. **c**. After more than three weeks of antibiotic treatment, the wound of the lateral drainage tube healed without effusion

The patient was treated with incision, debridement and irrigation of the left knee on December 15th. Intraoperatively, the knee cavity was filled with purulent, bloody liquid, the synovium was moderately hypertrophic and hyperemic, and there was severe inflammatory tissue hyperplasia on the articular surface of the anterior femoral condyle and joint space, but the cartilage appeared normal (Fig. 2.a). Suction, resection and repeated irrigation with iodophors, hydrogen peroxide and normal saline in the knee joint were performed thoroughly, and 2 flushing tubes and 2 suction drainage tubes were placed in the knee joint. Postoperatively, an antifungal regimen was carried out.

After a week of intravenous voriconazole and knee irrigation with amphotericin B, a recheck of the inflammatory markers revealed that the erythrocyte sedimentation rate was 28 mm/h, and the level of high-sensitivity C-reactive protein was 8.5 mg/dL. Then, we discontinued the knee irrigation. Two days later, yellow purulent liquid was drained out in the medial drainage tube, and the tube became obstructed (Fig. 2.b). Considering the poorly controlled infection and the possibility of knee joint adhesion, a secondary incision, debridement and irrigation of the left knee was performed on December 27th. The intraoperative findings were similar to the first operation. A tissue sample was collected for culturing. The results of bacteria culture and drug sensitivity tests were negative, so the antifungal regimen and knee joint irrigation with amphotericin B were continued.

On January 6th, 2017, the skin around the drainage tube on the medial side of the left knee was a mild red color and swollen, the laboratory examination revealed an obviously elevated erythrocyte sedimentation rate (44 mm/h) and elevated levels of high-sensitivity C-reactive protein (150.9 mg/dL). The drainage tubes were unobstructed and smooth. Considering that the elevation of the inflammatory markers might be associated with a cellulitis, IV clindamycin hydrochloride (0.45 g, q8 h) was administered for a week, and then IV clindamycin hydrochloride was discontinued and replaced with clindamycin palmitate hydrochloride dispersible tablets (150 mg PO qid). The medial flushing tube and drainage tube were removed, but the result of the bacteria culture of the skin sample was negative.

On January 11th, after almost a month of the antifungal regimen, the blood examination was repeated to analyze hepatic function and inflammatory markers. The results revealed that the erythrocyte sedimentation rate (29 mm/h) and high-sensitivity C-reactive protein concentration (19.5 mg/dL) were lower than those observed during the previous examination result. The left knee joint irrigation was discontinued, intravenous voriconazole was also discontinued, fluconazole 400 mg IV q24 h was initiated, and the lateral flushing tube and drainage tube were maintained to continue draining the joint liquid. After two weeks of antibiotic treatment, the operative wound of the lateral drainage tube slightly effused a light-yellow liquid, so the liquid sample was sent for culture, and the result revealed *Pseudomonas aeruginosa* and *Enterobacter cloacae* infections. According to the drug sensitivity tests, clindamycin palmitate hydrochloride dispersible tablets were discontinued and replaced with ciprofloxacin lactate (0.2 g IV q12 h). After another week of antibiotic treatment, the symptoms were noticeably improved, the knee joint had no swelling, and the operative wounds were healing without effusion but had some sligh pain (Fig. 2.c), and the joint was able to be moved well.

**Fig. 3:**
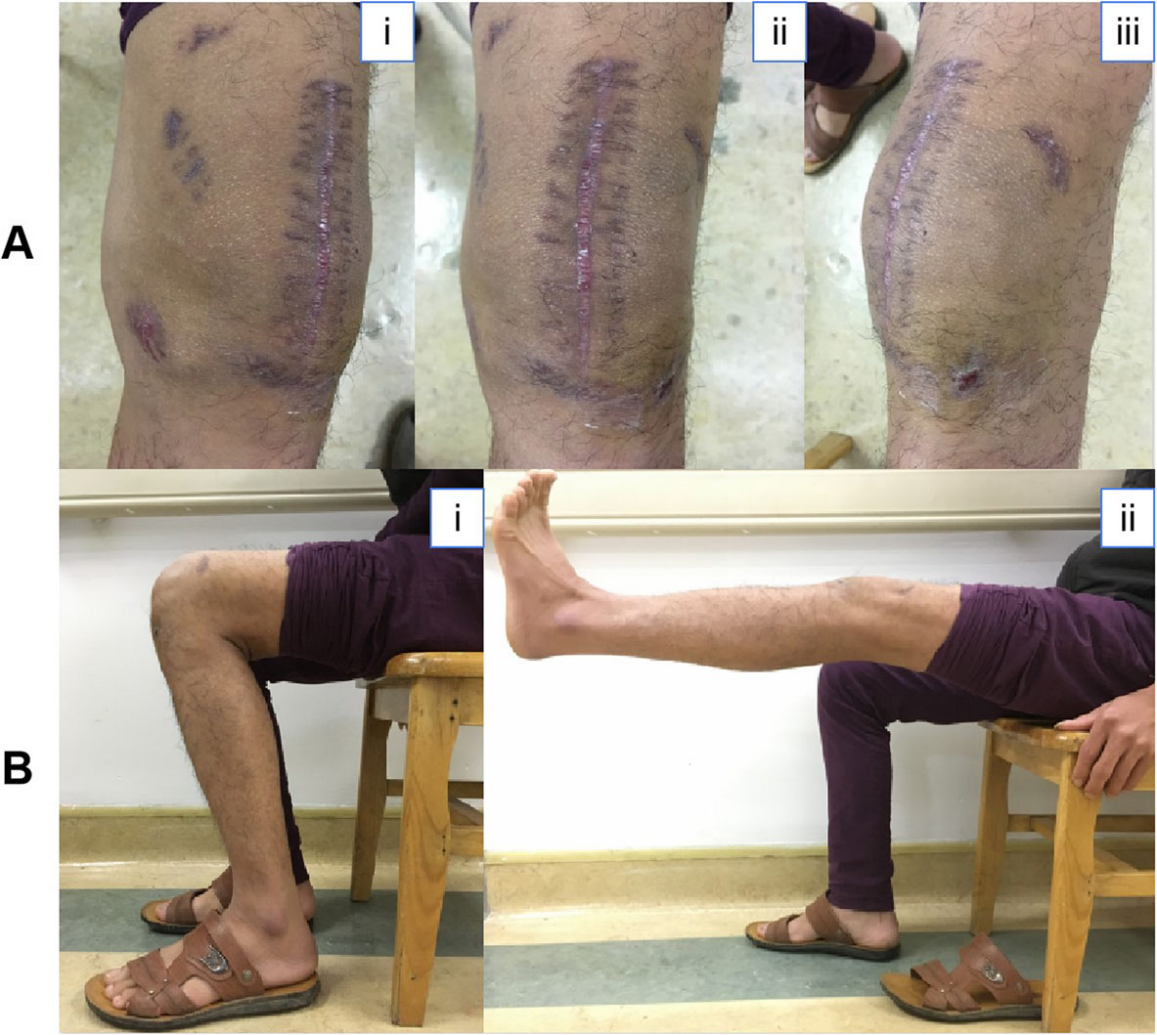
**a**.Two months later, a physical examination showed no swelling, pain or warmth of the left knee; the medial, middle and lateral operative wounds were healed well; and the infection did not recur. **b**.Three months later, the left knee was painless, and the range of motion of the left knee was 0–120°

The patient was discharged on January 30th, 2017, and an antifungal and antibacterial therapy of fluconazole (400 mg PO qd) and ciprofloxacin lactate were continued for 6 months and 3 weeks, respectively. Three months later, a physical examination showed that there was no swelling, pain or warmth of the left knee; the medial, middle and lateral operative wounds were healed well with no infection recurrence; and the range of motion of the left knee was 0–120° (Fig. 3.a and b).The patient is very satisfied with the curative effect. See Table 1 for details of the schematic representation of the timeline.

**Table 1:** Timeline

Timeline	Clinical manifestations / Examination results	Treatment
March 2016	Stabbed by plants on the posterolateral side of his left knee.Pain, warmth and swelling in the left knee joint.	Antibiotics, intra-articular puncture with knee fluid aspiration and steroidal injection.
November 4th, 2016	Admission of patient. The left knee was moderately swollen without effusion and the local skin temperature was slightly increase.	
November 7th, 2016		Surgical of the left knee with a synovectomy and meniscus repair under arthroscopy. Postoperatively, the left knee was irrigated with Gentamicin sulfate and other routine treatments were carried out for 5 days.
November 15th, 2016	The patient’s symptoms were relieved and discharged.	
November 23th, 2016	Culture result of the patient’s operative sample revealed a *Cryptococcus laurentii* infection.	Inform the patient to return to the hospital for treatment.
December 12th, 2016	Admission of patient.Left knee was moderately swollen and warm, and more than 5 ml of yellow purulent fluid was withdrawn	200 mg of intravenous (IV) voriconazole every 12 h (q12 h; 400 mg q12 h on the first day), debridement and irrigation of the left knee with amphotericin B until the infection was controlled and 400 mg of fluconazole per os (PO) q24 h maintained for 6 months.
December 15th, 2016		Incision, debridement and irrigation of the left knee. Suction, resection and repeated irrigation with iodophors, hydrogen peroxide and normal saline in the knee joint were performed thoroughly, and 2 flushing tubes and 2 suction drainage tubes were placed in the knee joint. Postoperatively, an antifungal regimen was carried out.
December 27th, 2016	The tube became obstructed.	Secondary incision, debridement and irrigation of the left knee was performed.The antifungal regimen and knee joint irrigation with amphotericin B were continued.
January 6th, 2017,	The skin around the drainage tube on the medial side of the left knee was a mild red color and swollen. ESR:44 mm/h, hsCRP:150.9 mg/dL.	IV clindamycin hydrochloride (0.45 g, q8 h) was administered for a week, and then IV clindamycin hydrochloride was discontinued and replaced with clindamycin palmitate hydrochloride dispersible tablets (150 mg PO qid).
January 11th, 2017,	ESR:29 mm/h, hsCRP:19.5 mg/dL.	The left knee joint irrigation was discontinued, intravenous voriconazole was also discontinued, fluconazole 400 mg IV q24 h was initiated, and the lateral flushing tube and drainage tube were maintained to continue draining the joint liquid.
January 25th, 2017,	The operative wound of the lateral drainage tube slightly effused a light-yellow liquid, so the liquid sample was sent for culture, and the result revealed *Pseudomonas aeruginosa* and *Enterobacter cloacae* infections.	Clindamycin palmitate hydrochloride dispersible tablets were discontinued and replaced with ciprofloxacin lactate (0.2 g IV q12 h).
January 30th, 2017	No swelling, and the operative wounds were healing without effusion, and the joint was able to be moved well.	Discharged from hospital.Antifungal and antibacterial therapy of fluconazole (400 mg PO qd) and ciprofloxacin lactate were continued for 6 months and 3 weeks.
April, 2017	Outpatient follow-up.There was no swelling, pain or warmth of the left knee; the medial, middle and lateral operative wounds were healed well with no infection recurrence; and the range of motion of the left knee was 0–120°	

## Discussion and conclusion

Human cases of *Cryptococcus laurentii* infections in various organs and systems have been reported, including *Cryptococcus laurentii* fungaemia and *Cryptococcus laurentii* diarrhea in cancer patients [11, 12], *Cryptococcus laurentii* meningitis in HIV patients [13] and other reports of skin and eye infections [14, 15].

In general, the treatment includes surgical removal of necrotic tissue and removal of potentially infected implants, combined with long-term antifungal therapy. It is necessary to evaluate the immune status of patients accurately. Although the common infection can be cured by antifungal therapy alone, the infection after surgery has its particularity. If there is no surgical debridement, it is difficult to achieve successful results [16]. Antifungal drugs should be determined under the guidance of pathogen identification and antifungal sensitivity. We summarized the treatment guidelines for various fungal infections for clinicians’ reference [17–23] (Table 2).

**Table 2:** Summary of Infectious Diseases Society of America guidelines for the treatment of fungal osteoarticular infections

Category	Treatment	Comments
*Cryptococcus neoformans*	Osteoarticular infections are not specifically addressed in current IDSA guidelinesRecommendations for extrapulmonary non-CNS cryptococcosis in immunocompetent patients: follow treatment protocol for CNS disease (see guidelines for separate recommendations for HIV-positive patients and for transplant recipients)Induction therapy:1. AmB plus flucytosine for 4 wk.2. AmB for 6 wk.3. Liposomal AmB or ABLC combined with flucytosine, if possible, for 4 wk.; or4. AmB plus flucytosine for 2 wk. (for patients at low risk for therapeutic failure; see guidelines)Consolidation therapy: fluconazole (400–800 mg/d) for 8 wk.Maintenance therapy: fluconazole (200 mg/d) for 6–12 moPatients without cryptococcemia and with a single site of infection and no immunosuppressive risk factors:Fluconazole for 6–12 moDepending on immune status, patients may require long-term secondary prophylaxis with fluconazole	
*Candida* ^sp^	Fluconazole, or Echinocandin (caspofungin, micafungin, or anidulafungin) for at least 2 wk. followed by fluconazole, or Liposomal AmB for at least 2 wk. followed by fluconazole	Choice of antifungal agent should be guided by susceptibility testingDuration of treatment:Septic arthritis: 6 wk.Osteomyelitis: 6–12 mo for osteomyelitis
*Aspergillus* ^sp^	Primary: voriconazoleAlternative: liposomal AmBSalvage:ABLCCaspofunginMicafunginPosaconazoleItraconazole	Duration: minimum of 8 wk., to > 6 mo Guidelines recommend following same treatment protocols described for invasive pulmonary aspergillosis, but note that there is little experience with echinocandins in the treatment of aspergillus OAI
*C immitis*	Mild-moderate disease: fluconazole or itraconazole Severe disease: liposomal AmB for 3 mo followed by fluconazole or itraconazole	Duration of therapy: 3 y to lifelong
*Histoplasma capsulatum*	Mild-moderate disease: itraconazole Severe disease: liposomal AmB for 2–6 wk., followed by itraconazole	Histoplasma osteoarticular infections usually occur in the setting of disseminated disease. Duration of therapy for disseminated disease: at least 12 mo
*B dermatitidis*	Mild-moderate disease: itraconazole Severe disease: liposomal AmB for 2 wk. followed by itraconazole	Recommended duration of therapy for osteoarticular disease: at least 12 mo
*Sporothrix schenckii*	Preferred: itraconazole Alternative: liposomal AmB with change to itraconazole after a favorable response is achieved	Recommended duration of therapy for osteoarticular disease: at least 12 mo

In our patient, there were two risk factors that may have enhanced the index of suspicion of fungal infection upon first admission in our institution. One was that the patient received a penetrating wound from a thorn on the posterolateral side of his left knee, and they could be the cause of fungal infection. *Cryptococcus laurentii* can be isolated from a wide variety of environments, including air, water, wood, soil, pigeon excreta and many kinds of food [5]. David Chen-Guan Ong et al. reported a similar case about a right knee infection of fungus Arthrographis kalrae that was related to a penetration history [11]. The other risk factor was that the patient was treated several times was steroid injections before admission. Intra-articular steroid injection is a risk factor of infection. An outbreak of fungal infection due to contaminated steroid injections in United States has been reported before [12]. Based on the history of a penetrating knee wound and the lack of evidence of a problem with contaminated steroids during this timeframe in China, the authors believe the penetrating wound to be the more likely route of exposure.

The clinical manifestations of fungal infection are various, which are closely related to the types of fungi, infection sites and the health status of patients. The diagnosis of fungal infection is usually difficult, which requires many times of culture and identification. Thus, a broad array of sample cultures of samples from the knee, including fungi, tuberculosis and other bacteria, were performed after the surgery. The results of the cultures were negative, and the patient’s symptoms improved a lot after synovectomy and meniscus plasty surgery, so that we let him discharge temporarily.

After the second admission, a consult with a pharmacy specialist was held immediately to determine the antifungal regimen. Cooperation with the pharmacy specialist was necessary to treat the fungal infection, and other authors have also emphasized the importance and necessity of cooperation with pharmacy specialists or infection scientists. Thorough debridement is considered pivotal; we performed debridement surgery twice to clear the fungal infected tissue. We decided to perform the second one when we found that the irrigation tube was obstructed after over a week of antifungal treatment. Therefore, repeated, timely and effective debridement are important, and the patient should be informed of the possibility of repeated surgery beforehand. The second experience is that the course of irrigation treatment should be adequate. Although this is the first report of *Cryptococcus laurentii* infection of knee, and the proper treatment for the type of *Cryptococcus laurentii* infection was not yet established, we propose that local irrigation can be a pivotal choice to control the local infection, as our patient received nearly a month of irrigation with amphotericin B. After nearly a month treatment of antifungal drugs and debridement, twice the patient’s skin around the irrigation tube became red and slightly swollen. We considered that may have revealed a local skin bacterial infection, so an empiric antibiotic was used, and the effusion of the knee wound was sent to culture. The results confirmed our inference, and we adjusted the antibacterial regimen in time, according to the susceptibility.

In conclusion, we report a case of *Cryptococcus laurentii* knee infection in a previously healthy patient. To our knowledge, osteoarticular *Cryptococcus laurentii* infection has never been reported so far, and this is the first report of knee *Cryptococcus laurentii* infection. The patient received nearly a month of voriconazole (200 mg IV q12 h) and knee irrigation with amphotericin B until the infection was controlled, and the treatment was continued with fluconazole for nearly 7 months, with approximately 5 weeks of antibiotic treatment for the skin bacteria coinfection. The patient’s symptoms disappeared, the knee recovered well, and no infection recurred. We propose that the key points of successful treatment in this case were the thorough debridement, the adequate course of knee irrigation with antifungal drugs and more than 6 months of oral antifungal drugs that were able to eradicate the infection.

## Data Availability

This article is a case report. Data and imaging data are provided in this article. And the other more data can be requested from the corresponding author.

## Abbreviations

IV: Intravenous injection
PO: Per os
MR: Magnetic resonance
HIV: Human immunodeficiency virus
PMMA: Polymethyl methacrylate
